# Pneumococcal Meningitis

**DOI:** 10.5334/jbsr.2993

**Published:** 2022-12-29

**Authors:** Kelly Di Dier, Marc Lemmerling

**Affiliations:** 1 AZ Sint-Lucas Ghent, BE

**Keywords:** pneumocephalus, meningitis, computed tomography, magnetic resonance imaging, neuroradiology

## Abstract

**Teaching Point:** Spontaneous, atraumatic pneumocephalus is a rare presentation of pneumococcal meningitis.

## Case History

**Figure 1:**
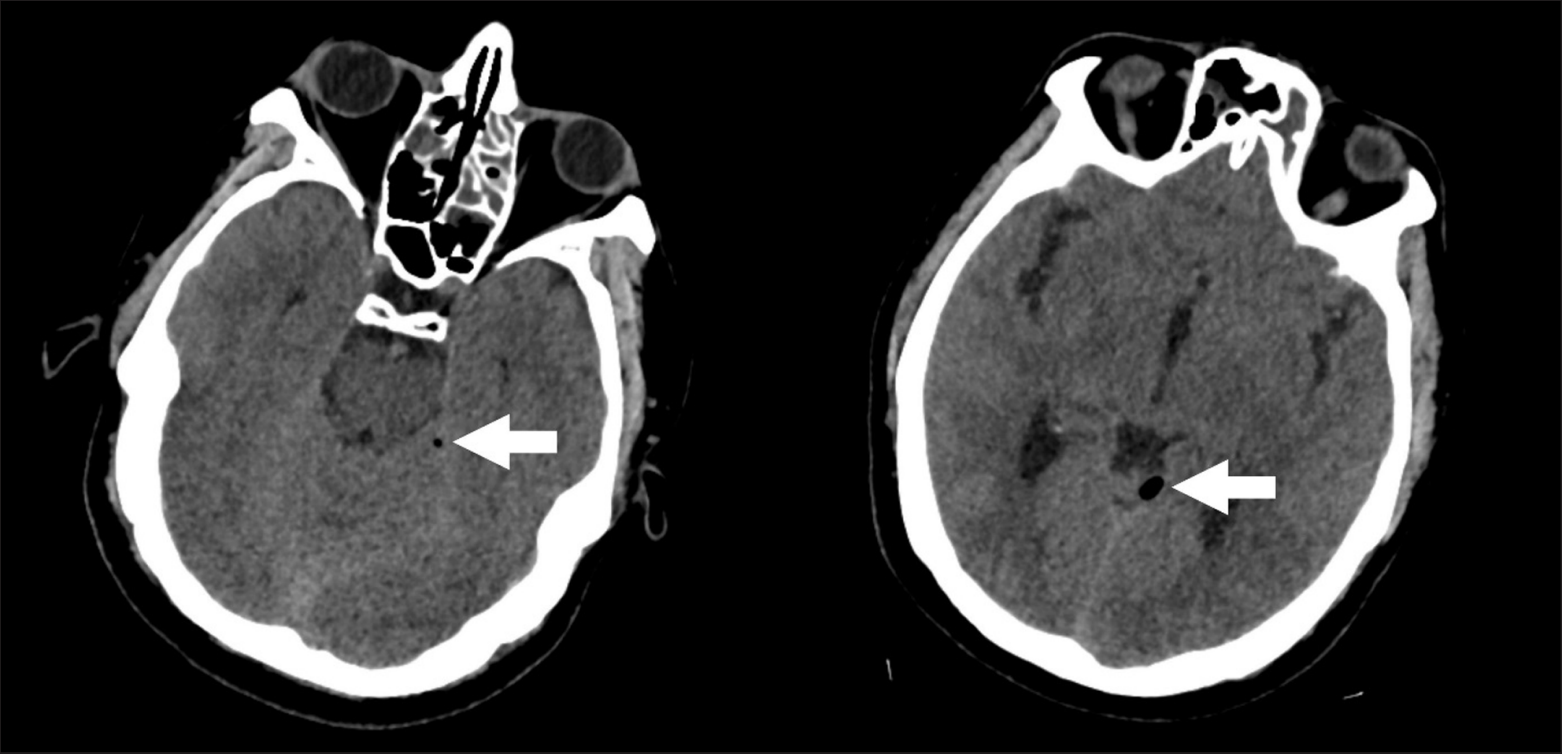


**Figure 2:**
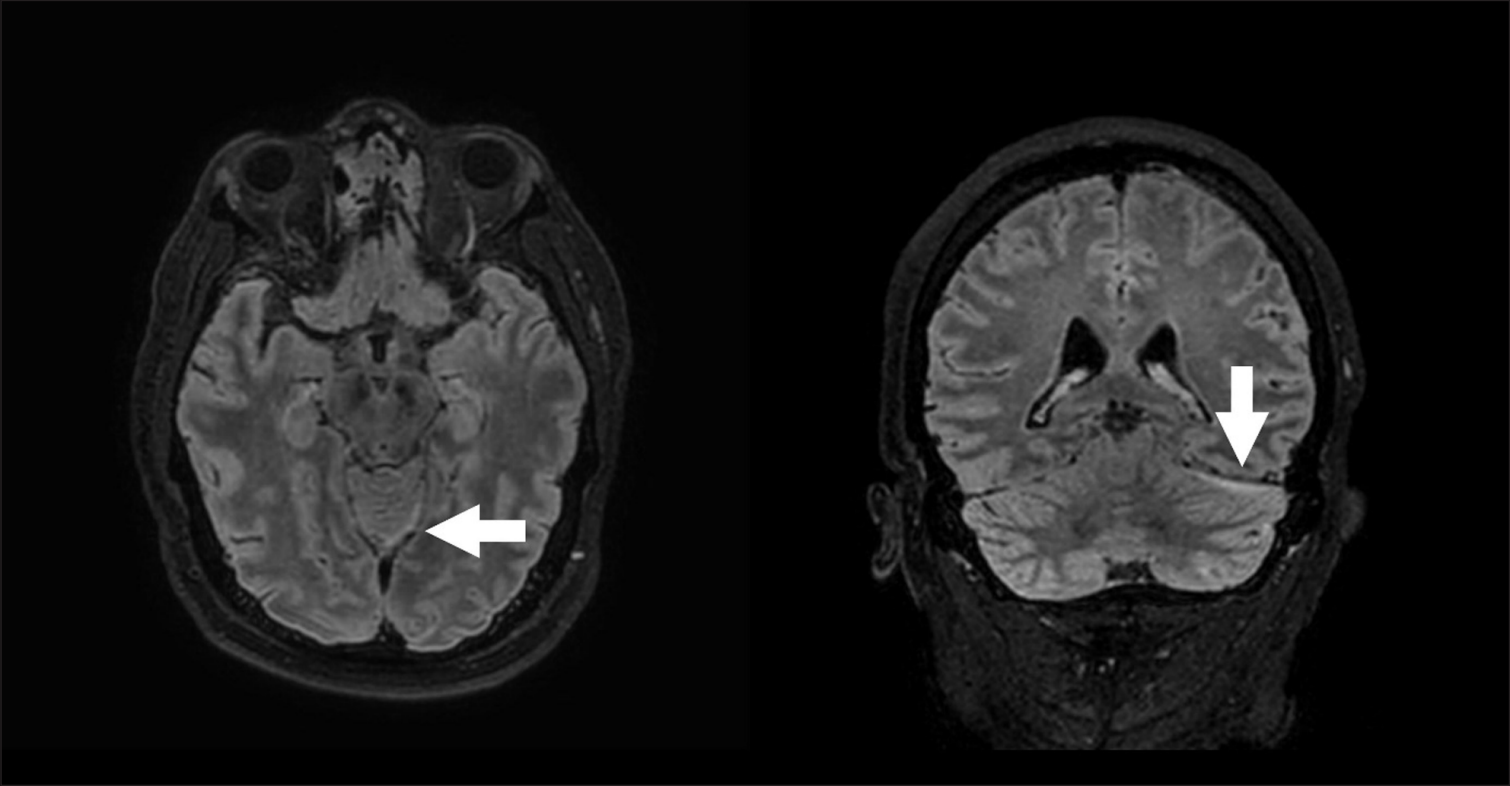


**Figure 3:**
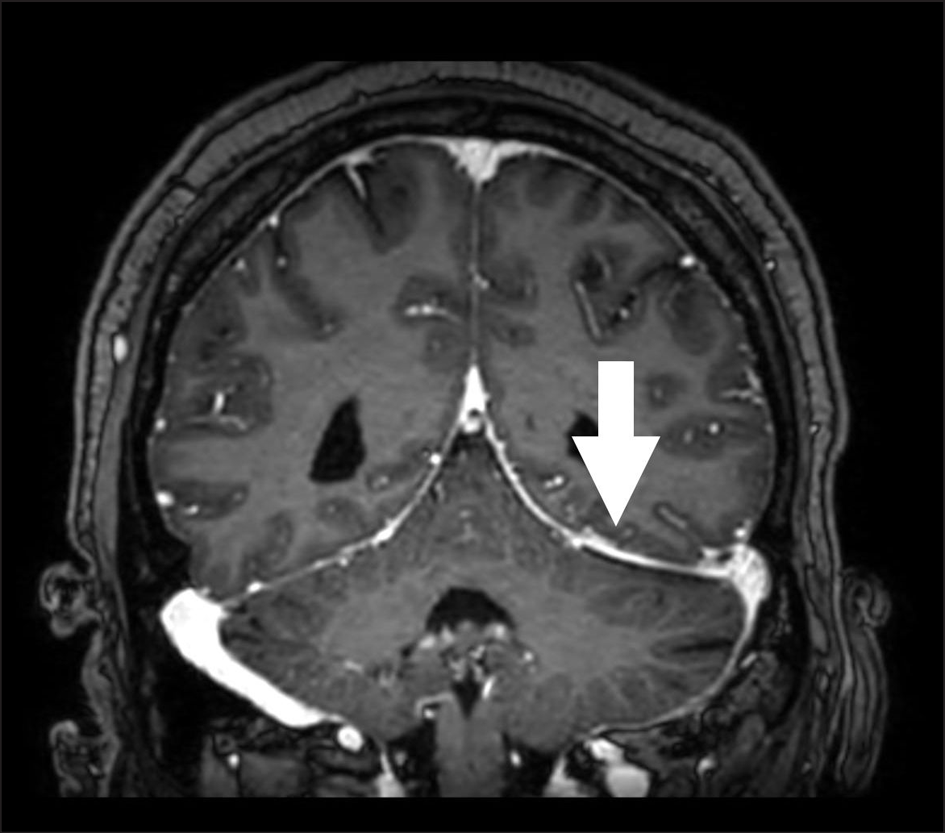


A 43-year-old male presented at the emergency department with atraumatic increasing headache, nausea and vomiting. Neurological examination revealed neck stiffness, photo- and phonophobia. Subsequent non-contrast enhanced computed tomography (CT) of the brain was executed, showing two subtle air bubbles adjacent to the left-sided tentorium cerebelli (Figure 1). Subsequent magnetic resonance imaging (MRI) of the brain was performed. On fat-suppressed 3D FLAIR-weighted images, the meninges on the left side of the tentorium cerebelli appeared asymmetrically hyperintense compared to the right side (Figure 2). Subtle increased contrast enhancement of these meninges was noted on T1-weighted images after intravenous contrast administration (Figure 3), suggestive of meningitis. Lumbar puncture was performed of which the collected cerebrospinal fluid culture detected Streptococcus pneumoniae, confirming the diagnosis of pneumococcal meningitis.

## Comments

Pneumocephalus is a condition where intracranial gas is present and occurs frequently in a traumatic or postoperative setting. Atraumatic pneumocephalus is rather rare and should raise the suspicion of meningitis with gas producing bacteria, such as pneumococcus [1]. Multiple symptoms are associated with meningitis, such as headache, neck stiffness, nausea, vomiting, and fever.

When bacterial/pyogenic meningitis is present, the damaged cells die. Intracellular components, such as proteins and glucose, will be decomposed. In this process of decay, gas is produced. Pneumocephalus is most easily detected on a CT of the brain, as this technique is capable of showing very small gas bubbles. Note that the pneumocephalus will resolve quite quickly, frequently during the first week. Therefore, it is solely observed in an early disease stadium [1].

MRI of the brain can contribute to the suspected diagnosis of meningitis. FLAIR-weighted images can reveal hyperintense subarachnoid areas, following the sulci/meningeal contour. This is well demonstrated in Figure 2. Enhancement after intravenous contrast administration is noted in the affected areas.

The radiological examinations should always be completed with a lumbar puncture. The obtained cerebrospinal fluid will be cultured, in order to detect growth of pathogens. Multiple pathogens can cause meningitis: viruses, bacteria, mycobacteria and fungi. The treatment will be adjusted according to the causative pathogen. Pneumocephalus is rather specific for an underlying bacterial infection. If bacteria generated the meningitis, the treatment will be composed out of antibiotics.

Meningitis with pneumocephalus can have a severe disease course, though it is not necessarily fatal. Babies and young infants are most at risk to die from pneumococcal meningitis.

In conclusion, when atraumatic pneumocephalus occurs in the emergency setting, underlying pneumococcal meningitis should be suspected.
